# Improving HIV-1 tropism determination by combining geno2pheno and V3 net charge calculation

**DOI:** 10.7448/IAS.15.6.18215

**Published:** 2012-11-11

**Authors:** C Montagna, E De Crignis, O Turriziani, I Bon, M Re, G Antonelli, G Pantaleo, C Graziosi

**Affiliations:** 1 University of Rome, Roma, Italy; 2 Centre Hospitalier Universitaire Vaudois, Lausanne, Switzerland; 3 University of Rome “La Sapienza”, Roma, Italy; 4 University of Bologna, Bologna, Italy

## Abstract

Genotypic tests are the most common methods to identify patients eligible for CCR5 inhibitors administration in Europe. Among the available tools geno2pheno coreceptor (G2P) is the most used online system in routine diagnostics. This study was conceived to assess if the combination of G2P prediction with V3 peptide net charge (NC) value could improve the accuracy of tropism prediction. Sequences (129) were analyzed by G2P according to European Guidelines. NC values were calculated by the online software Peptide Property Calculator. Phenotypic assay was performed cloning the complete env gene into pcDNA 3.1 TOPO vector; infectivity of pseudotyped virions was tested on U87_CD4+CCR5+ and U87_CD4+CXCR4+ cells lines to assess viral tropism. Sequences were stratified into 3 groups according to the agreement between NC values and G2P results. Group 1: sequences assigned to the same group by both tools, group 2: sequences assigned to one group by G2P but indeterminate by NC and group 3: sequences for which G2P and NC gave discordant results. 61% of sequences predicted as X4 by G2P showed NC values higher than 5; similarly, 76% of sequences predicted as R5 by G2P had NC values below 4 (Group 1). Sequences with NC values between 4 and 5 (Group 2) were associated to different G2P predictions: 59% samples were predicted as R5-tropic and 41% sequences as X4-tropic. These data support the hypothesis that 4 to 5 NC values could be associated to the presence of dual/mixed-tropic variants (DM). Sequences identified as X4 by NC value had at least one positive residue in positions known to be involved in tropism prediction (58%) and positive residues in position 32 (39%). To further verify NC-based prediction, phenotypic assay was performed on a subset of sequences from each group. The assay confirmed the tropism prediction for group 1 sequences and demonstrated that the variants with net charge between 4 and 5 have DM tropism. Moreover, in vitro phenotyping of discordant viruses confirmed NC result, showing that this parameter is strongly associated with phenotypic assay. These results show that the combination of G2P and NC could increase the accuracy of tropism prediction and the ability to discriminate DM viruses. A more reliable identification of X4 variants would be useful for better selecting candidates for maraviroc administration, but also as a predictive marker in coreceptor switching, strongly associated to the phase of infection.

